# Mixed cumulative probit: a multivariate generalization of transition analysis that accommodates variation in the shape, spread and structure of data

**DOI:** 10.1098/rsos.220963

**Published:** 2023-03-01

**Authors:** Kyra E. Stull, Elaine Y. Chu, Louise K. Corron, Michael H. Price

**Affiliations:** ^1^ Department of Anthropology, University of Nevada, 1664 North Virginia Street, Stop 0096, Reno, NV 89557, USA; ^2^ Forensic Anthropology Research Centre, Department of Anatomy, University of Pretoria, Private Bag x323, 0007 Pretoria, South Africa; ^3^ Complexity Nexus LLC, Pittsburg, PA, USA

**Keywords:** Bayesian statistics, information theory, heteroscedasticity, conditional dependence, age estimation, subadult

## Abstract

Biological data are frequently nonlinear, heteroscedastic and conditionally dependent, and often researchers deal with missing data. To account for characteristics common in biological data in one algorithm, we developed the mixed cumulative probit (MCP), a novel latent trait model that is a formal generalization of the cumulative probit model usually used in transition analysis. Specifically, the MCP accommodates heteroscedasticity, mixtures of ordinal and continuous variables, missing values, conditional dependence and alternative specifications of the mean response and noise response. Cross-validation selects the best model parameters (mean response and the noise response for simple models, as well as conditional dependence for multivariate models), and the Kullback–Leibler divergence evaluates information gain during posterior inference to quantify mis-specified models (conditionally dependent versus conditionally independent). Two continuous and four ordinal skeletal and dental variables collected from 1296 individuals (aged birth to 22 years) from the Subadult Virtual Anthropology Database are used to introduce and demonstrate the algorithm. In addition to describing the features of the MCP, we provide material to help fit novel datasets using the MCP. The flexible, general formulation with model selection provides a process to robustly identify the modelling assumptions that are best suited for the data at hand.

## 1. Introduction

A statistical approach called transition analysis (TA) made a substantial impact in biological anthropology when it was introduced in 2002. TA is a statistical approach—or, more accurately, a set of similar approaches—within the broader family of ordinal response models. More precisely, it is a cumulative link generalized linear model [1–5]. It typically uses a probit link function and a probit regression to fit an intercept and slope to the interior term, which allows one to calculate the probability of each ordinal stage as a function of the outcome. Combined with a prior distribution over the outcome variable, one can then do such things as calculate the average change from one stage to the next (and the standard deviation for that transition) and calculate the posterior probability as a function of the outcome given a particular ordinal observation and prior age distribution. When there is more than one transition or, equivalently, when there are more than two stages, the model is a cumulative probit. TA could theoretically use any ordinal trait to estimate any continuous outcome; however, in biological and forensic anthropology, TA is overwhelmingly associated with age estimation. While it has been used in subadult age estimation (e.g. [6–8]), it is more commonly associated with adult age estimation using the pubic symphysis, auricular surface and cranial sutures (i.e. ADBOU) [1,3,9–11].

In this article, we describe a novel algorithm, the mixed cumulative probit (MCP), that is a formal generalization of the single-variable cumulative probit model that underlies most TA models. We mean something quite precise by this: when the MCP is used to model a single ordinal variable with a linear mean response and homoscedastic noise response (concepts we describe below) one exactly recovers a cumulative probit model. The algorithm retains the underlying conceptual approach of TA, but with increased flexibility; for example, it accommodates continuous variables via a natural extension and allows nonlinear mean and noise responses. There is great practical benefit to the general approach we take because users need not decide beforehand which modelling assumptions are consistent with their data. The flexible, general formulation with model selection (e.g. via cross-validation in this paper) provides a process to robustly identify the modelling assumptions that are best suited for the data at hand.

The primary goal of this article is to provide a conceptual introduction to the MCP algorithm. The functions used to implement the algorithm are housed in a R package hosted on GitHub called yada, which stands for ‘Yet Another Demographic Analysis' (https://github.com/MichaelHoltonPrice/yada). Although we were motivated to develop the MCP by an interest in age estimation, the current intent is not to validate the MCP's utility as an age estimation model; a separate article will assess the performance of the MCP for subadult age estimation. The secondary goal is to provide a practical grounding for practitioners to use our algorithm. We provide both a static, fully reproducible pipeline or set of code to recreate the analyses presented in the manuscript (https://github.com/MichaelHoltonPrice/rsos_mcp_intro), and a vignette, which can yield fully reproducible results, but users can also easily implement the algorithm to answer their own research questions using the provided templates (RPubs.com/elainechu/mcp_vignette). The vignette describes each function and their arguments as one moves from univariate to multivariate models. There are copious comments and documentation in the code, and, furthermore, the greater than 1000 tests exercise all the functionality of the yada R package and provide example code that can be modified.

### 1.1. The impetus

The Bayesian framework of TA provides a number of advantages over frequentist, regression-based approaches, such as the reduction of downstream consequences of age mimicry (i.e. age-at-death distributions mimic those of the reference sample on which age estimation methods were based) [1,3,12]. The probabilistic nature of Bayesian approaches captures the relationship between prior beliefs (prior) and the inclusion (or exclusion) of evidence (likelihood). Specifically, the Bayesian approach calculates the posterior probability distribution, which is the likelihood (evidence present) times the prior (prior beliefs) divided by the normalization constant (evidence regardless of other dependence). The posterior probability distribution provides a formal quantification of uncertainty that has the capacity to be updated with new information (via the likelihood and prior). Therefore, Bayesian approaches are considered the most appropriate for assessing scientific evidence inside and outside of anthropology [13–20].

One component of a Bayesian approach that is challenging and debated is the elicitation of the prior, or the probability distribution that represents the uncertainty about the parameter prior to examining the evidence. The complexities of the prior have been discussed at length in forensic settings when the target population is not known (e.g. [17,21]). An uninformative prior assumes, in the case of age estimation, that all ages at death are equally likely. In contrast with this, an informative prior can be derived from any source with available background information. For example, with age estimation, this would be a distribution of ages at death in a population [19,21–23]. Informative priors yield more accurate age estimates compared with using an uninformative/uniform prior because they in fact provide useful information [21,24].

TA has been used for a myriad of research endeavours in biological anthropology (e.g. [6–8,13,17,21,25–34]). There has been an impressive shift from simple models to multi-variable and/or multi-factorial models (e.g. [29,34–41]). Even though TA models have progressed to multivariate or multi-factorial models, they have been restricted to ordinal input variables. The only exception we are aware of is provided by Gueorguieva & Agresti [42] who describe a cumulative probit model consisting of a single binary and a single continuous variable (i.e. a mixed model). For clarity, all mixed models are multivariate, but not all multivariate models are mixed. However, to our knowledge their model has never been used in biological anthropology, let alone age estimation.

Notable non-TA age estimation models can accommodate a mix of variable types, such as multivariate adaptive regression splines [43–45], but these are mostly regression models in a frequentist framework. Regression models have some inherent failings and, ultimately, may be more restrictive than useful in biological anthropology [41]. Biological data rarely fits the assumptions of linearity (shape) and homoscedasticity (spread). Importantly, the shape and the spread of the data are used to quantify uncertainty around the predicted ages (i.e. 95% prediction interval), and therefore, if the core assumptions are not met, the downstream reporting of the uncertainty may not be valid. Subsequently, anthropologists will transform the data or subset the data according to changes in slopes or in the spread of variance to fit the assumptions of the chosen statistical analyses. Examples of this can be seen throughout the human biology and biological anthropology literature, whether the analyses are conducted in bioarchaeology, forensic anthropology, demography or allometry (e.g. [43,44,46–55]).

When data are nonlinear, present with a non-normal distribution, or are heteroscedastic, transformations can be implemented to mitigate the downstream impacts [56,57]. Even if the transformation(s) yield(s) a more suitable match to statistical modelling assumptions, the results of statistical tests performed on transformed data are often not relevant for the original, non-transformed data and subsequently may be difficult to interpret [56]. For example, if researchers log transform data and then compare the means, the lack of significance in the transformed data does not equate to a lack of significance in the original dataset. Furthermore, if using log-transformed data in linear regression analyses, the interpretation of the results requires downstream modifications to the coefficients. The appropriate interpretation of the results is dependent on what was modified, which could be the dependent variable, independent variable, or both variables.

Some researchers have dealt with nonlinearity by subsetting the data to fit two, or more, unique linear regression models (e.g. individuals above 2 years have a specific linear model and individuals below 2 years have a specific linear model) [48,49]. This is circular and counterproductive when used for age estimation since any division of an age estimation model into two or more ‘sub-models’ requires one to estimate (or guesstimate) an unknown's age prior to selecting the appropriate sub-model and then estimating age. Other researchers have implemented nonlinear regression methods [43–45], but still face issues with heteroscedasticity and subsequent interpretation.

Other components that are specifically difficult to deal with in regression models are missing data and mixes of ordinal and continuous data. More variables yield more information, but more variables also yield a higher propensity for instances of missing data. In anthropological research, missing data are usually considered to be the result of taphonomy, trauma or recovery rates. Less discussed, but just as important, is how missing data can be inherent to a subject and vary because of biological reasons, for example, differential growth and development trajectories. Using this example, missing data can be developmentally absent, which is informative compared with missing at random. This is an obvious issue if the data collection method starts at ‘first stage of appearance’ rather than ‘absence’. While seemingly trivial, the differential data collection strategies are apparent when visualizing missing data in subadult age indicators (electronic supplementary material, figure S4). For instance, in contrast with epiphyseal fusion data, approximately 50% of data are missing from the dentition in individuals younger than 10 years of age because it is not accounted for by developmental staging systems (electronic supplementary material, figure S4). Konigsberg *et al*. [38] incorporated a ‘crypt absent’ and ‘crypt present’ stage, which yields fewer missing data for younger individuals. Another instance of missing data is when age indicators transition into non-age indicator variables. For example, as long bones increase in length during active growth, they are considered an age indicator for subadults. However, as soon as epiphyseal fusion is active, the length of the bone is no longer considered an age indicator. Fusion stages of proximal and distal long bone epiphyses, which indicate active maturation, are used instead. These examples are quite specific to growth and development, but conceptually are important to consider because of how cumulative probits result in average ages of transition between ordinal stages.

The preceding review of anthropological and statistical literature makes it abundantly clear that a Bayesian framework is best for modelling biological data; however, no algorithm is currently available that can accommodate common features of biological data. The MCP was developed to accommodate, in a single algorithm, multiple statistical characteristics of the underlying data that are empirically salient. Specifically, the MCP accommodates heteroscedasticity, ordinal and continuous response variables, missing values, conditional dependence and alternative specifications of the mean response (shape) and noise response (spread). Additionally, we used the Kullback–Leibler (KL) divergence statistic to quantify the severity of model mis-specifications, such as incorrectly assuming conditional independence. Importantly, although we developed the MCP algorithm for use in biological anthropology, the algorithm is applicable to any situation with the same data structure and modelling needs. Therefore, this algorithm could be used to estimate adult age at death, stature and time since death, among other topics, as well as being used beyond the boundaries of biological anthropology.

## 2. Materials and methods

The data used are part of the Subadult Virtual Anthropology Database (SVAD), a database comprising growth and development markers of geographically diverse children aged between birth and 22 years [58]. Data were primarily collected from computed tomography (CT) images generated in the past approximately 10 years, which offered the ability to collect up to 64 variables on each individual. The variables currently available in SVAD include 18 measurements of the six long bones (humerus, radius, ulna, femur, tibia and fibula), dental developmental stages of the 32 permanent teeth, and epiphyseal fusion stages of the proximal and distal long bone epiphyses, carpals, tarsals, the patella, the calcaneal tuberosity, the ilium and the ischium. The magnitude of data (both in the number of samples and the number of variables) is a remarkable feature of ‘virtual samples’ and provides an extreme advantage when compared with ‘classic’ skeletal collections comprising solely physical remains. As such, we were able to develop a model using a high-dimensional data frame with coverage across the growth and development period.

For the data-driven and visual explanation of the MCP, we included individuals between the ages of birth and 22 years from the United States. The sample (*n* = 1296) is composed of individuals from two medical examiner's offices in the United States: University of New Mexico Health Sciences Center, Office of the Medical Investigator (*n* = 1053, 81% of total) and the Office of the Chief Medical Examiner in Baltimore, Maryland (*n* = 243, 19% of total). Because the purpose of the manuscript is to describe the algorithm and illustrate its capacities and not discuss the performance of the resulting age estimation models, we chose to randomly sample six of the 64 variables in the SVAD, while retaining an even representation of the variable types (continuous, binary and ordinal). The six variables randomly chosen for inclusion are femoral diaphyseal length (FDL), radius diaphyseal length (RDL), humerus medial epicondyle epiphyseal fusion (HME_EF), tarsal ossification (TC_Oss), mandibular lateral incisor development (man_I2) and maxillary first molar development (max_M1). The two diaphyseal lengths were taken to the nearest hundredth of a millimetre on three-dimensional surface reconstructions of the corresponding skeletal elements following definitions for virtual elements. The four ordinal variables were scored directly on CT scans of the individuals using three different scales [59]. Epiphyseal fusion for the humeral medial epicondyle (HME_EF) was collected using a seven-stage system but collapsed into a four-stage system of absent (0), present (1), active fusion (1/2, 2, 2/3, 3) and fused (4) [60]. The number of tarsal bones (TC_Oss) present ranged from 0 (none present) to 7 (all present) though similar appearance times led to collapsing into six stages (calcaneus and talus were collapsed). Dental development (man_I2 and max_M1) was scored using a 13-stage system ranging from 1 (initial cusp formation) to 13 (apex closed) [61], though the last two apical stages were collapsed. All data were collected through the Amira™ three-dimensional visualization and reconstruction software (Amira™ v. 6.7.0. 1999–2018 FEI SAS, a part of Thermo Fisher Scientific), and detailed methodologies for all variables and their associated error rates can be found in the SVAD data collection protocols [59,60] and in Corron *et al*.'s study [62].

### 2.1. The algorithm

Below is a high-level, conceptual introduction to the MCP algorithm. A complete description of the model is provided in the electronic supplementary material, all source code to replicate the results are provided in a GitHub repository at GitHub.com/ElaineYChu/mcp_s-age_pipeline and a vignette providing the code on a step-by-step basis is provided at RPubs.com/elainechu/mcp_vignette. We begin by describing the special cases of single-variable (univariate) continuous and ordinal models, then discuss how cross-validation determines the best parametric specifications and how information theory helps with further interpretations. Next, we summarize our multivariate model, which accommodates mixtures of ordinal and continuous variables, as well as missing response variables. We also describe a second, distinct cross-validation step used to identify the conditional correlation structure that links variables and, again, how information theory helps with interpretations. Where appropriate, we refer the reader to figures and other results that underscore or reinforce the description of the statistical methods. All analyses were performed in the R statistical language [63].

#### 2.1.1. Univariate continuous models

**Figure 1.**
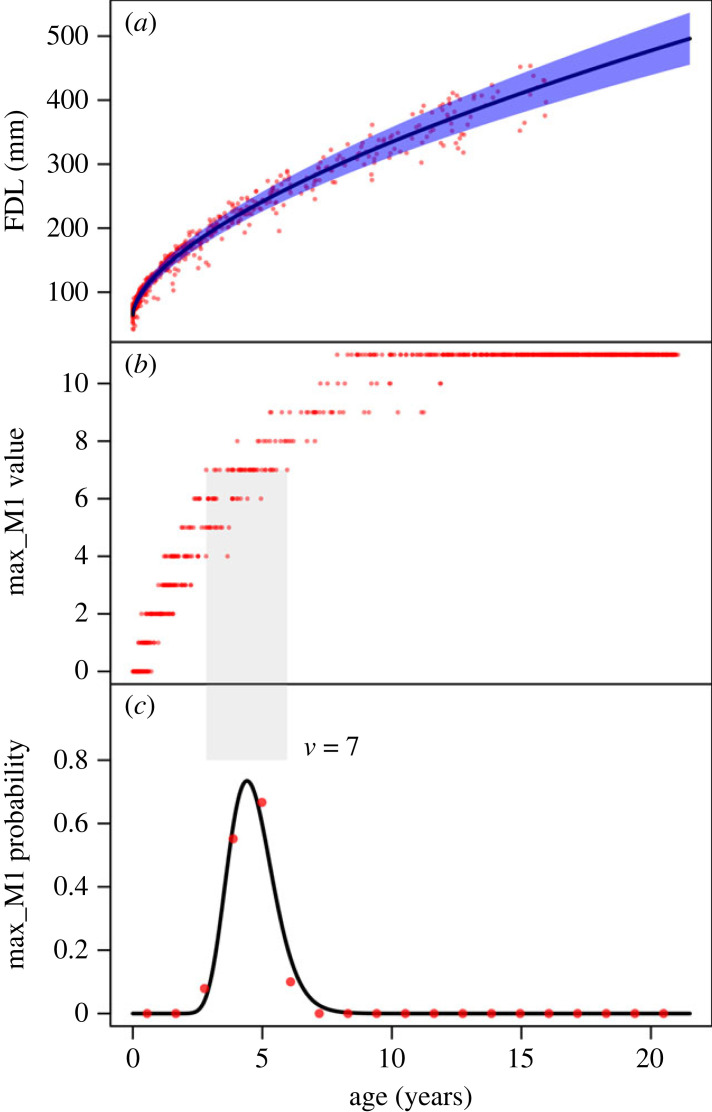
(*a*) FDL versus known age with a heteroscedastic maximum-likelihood fit. Red dots are observations, the black line is the mean response, and the blue shaded region marks the noise bounds. (*b*) Maxillary first molar (max_M1) developmental score versus known age. (*c*) Probability of observing the dental developmental stage of 7 (*v* = 7) as a function of age for max_M1. The grey band that extends from the middle to bottom plot marks the range of ages for which *v* = 7 is observed in the data. The black curve is the predicted probability the model preferred by cross-validation (power law for the mean and heteroscedastic noise). The red dots are the observed proportions in the underlying data, which are calculated by binning observations by known age value and calculating the proportion of observations in each bin for which *v* = 7.

For continuous variables, we assume that the observed response, *w* (the *y*-axis variable figure 1*a*), is normally distributed with a mean of h(x,c) and a s.d. of ψ(x,κ),

w ∼ N(h,ψ).

For the parametrization of the mean, we use a scaled, offset power law, $h(x,c)=c_{2}x^{c_{1}}+c_{3}.$ For the parametrization of the s.d., we consider either constant noise, $\psi (x,\kappa )=\kappa_{1}$ (homoscedastic) or linear positive noise, $\psi (x,\kappa )=\kappa_{1}[1+\kappa_{2}x]$ (heteroscedastic). To ensure that the standard deviation is always positive, we require all parameters to be positive; since the intercept for the heteroscedastic model must be positive, we refer to this noise model as ‘linear positive intercept.’ Figure 1*a* shows the FDL value (*w*) as a function of age (*x*). The red dots are pairs of values for individuals of known age. The solid line is the function h(x,c) that resulted from a maximum likelihood, univariate fit to the known age data for the heteroscedastic model. The shaded region shows the noise level as a function of age (h±ψ).

#### 2.1.2. Univariate ordinal models

For ordinal variables, it is the latent (unobserved) response that is normally distributed, with mean g(x,b) and s.d. γ(x,β),

$$v^{*}\;∼\;N(h,\gamma ).$$

The observed ordinal response, *v*, is related to the latent response, v*, via a vector of boundary parameters, τ, per

$$v=\{\begin{array}{c} 0\;\;\mathrm{if}\;-\infty <\;v^{*}\leq \tau_{1}\;\\ m\;\;\mathrm{if}\;\tau_{m}<\;v^{*}\leq \tau_{m+1}\\ M\;\;\mathrm{if}\;\tau_{M}<\;v^{*}\leq \infty \;\;\; \end{array}.$$

These boundary parameters, $\tau_{m}$, are part of the model parametrization and included in the maximum-likelihood fitting. The assumption that the noise is normally distributed makes this a probit model. This assumption of a probit link function is an entirely distinct assumption from the choice of the mean and noise responses. For statistical identifiability reasons (see electronic supplementary material, §1.3), we allow three specifications of the mean: (i) an unscaled, unshifted power law, $g(x,\;b)=x^{b_{1}}$; (ii) a logarithm, g(x, b)=log⁡x; or (iii) a linear function, g(x, b)=x. The response functions are unscaled and unshifted to ensure statistical identifiability (see electronic supplementary material, §1.3). Often, in previous work, one of these specifications of the mean is assumed without being checked, usually either the logarithm or the linear specification (e.g. [2,24]). We adopt the same noise specifications for ordinal variables as for continuous variables. In total, therefore, there are 3*2 = 6 distinct models that we cross-validate for univariate ordinal fits (see §2.13 and table 1). Figure 1*b* shows the ordinal value of max_M1 as a function of known age. It is more challenging to visualize ordinal fits; in figure 1*c* we visualize the fit for one ordinal response, *v* = 7, which refers to the dental developmental stage of 7.

**Table 1.** Variable information and the associated cross-validated results for Step 1 of the cross-validation (univariate models). The model with the smallest negative log-likelihood is considered the best fit (italicized). For each ordinal variable, six distinct models were assessed (three choices for the parametrization of the mean and two for the noise). For each continuous variable, two distinct models were assessed (one choice for the parametrization of the mean and two for the noise). The ‘constant’ noise specification is the homoscedastic model and the ‘linear positive intercept’ noise specification is the heteroscedastic model. The heteroscedastic model was preferred by cross-validation for five of the six models.

response variable	variable group	variable type	mean specification	noise specification	negative log-likelihood
humerus medial epicondyle (HME_EF)	epiphyseal fusion	ordinal	power law ordinal	constant	451.46
			*power law ordinal*	*linear positive intercept*	*451.04*
			logarithmic	constant	482.78
			logarithmic	linear positive intercept	482.78
			linear	constant	460.03
			linear	linear positive intercept	452.04
tarsal count (TC_Oss)	ossification	ordinal	power law ordinal	constant	441.14
			*power law ordinal*	*linear positive intercept*	*439.24*
			logarithmic	constant	rejected for mean specification not able to be fit
			logarithmic	linear positive intercept	rejected for mean specification not able to be fit
			linear	constant	498.58
			linear	linear positive intercept	452.79
maxillary first molar (max_M1)	dental development	ordinal	power law ordinal	constant	338.54
			*power law ordinal*	*linear positive intercept*	*335.88*
			logarithmic	constant	362.05
			logarithmic	linear positive intercept	362.05
			linear	constant	432.10
			linear	linear positive intercept	360.65
mandibular lateral incisor (man_I2)	dental development	ordinal	*power law ordinal*	*constant*	*352.97*
			power law ordinal	linear positive intercept	358.28
			logarithmic	constant	365.62
			logarithmic	linear positive intercept	365.62
			linear	constant	419.08
			linear	linear positive intercept	rejected for large beta2
FDL	long bone measurement	continuous	power law	constant	2464.90
			*power law*	*linear positive intercept*	*2352.01*
RDL	long bone measurement	continuous	power law	constant	1958.46
			*power law*	*linear positive intercept*	*1887.47*

#### 2.1.3. Cross-validation of univariate models (Step 1)

Visually, there is clearly greater variability in the FDL values of older individuals compared with younger individuals, which is reflected in the increasing value of noise as a function of age for the heteroscedastic fit (the shaded region in figure 1*a*). Similarly, there is greater variability in the max_M1 values of older individuals compared with younger individuals (figure 1*b*). Both visualizations suggest that a heteroscedastic noise model is needed. However, rather than relying on visual interpretations to choose parametric forms, we used fourfold cross-validation to determine, for each individual variable, the best parametric model, accounting for both the mean and noise responses. For clarity, researchers can use any number of folds or AIC to choose the parametric forms.

To accomplish this, we assigned each observation to one of four evenly sized test-folds, using the same cross-validation folds for all six variables. The remaining observations constitute the training data for that test fold. The vector of responses, ***y***, is for a single variable (and multiple individuals); it is equivalent to the vector of responses ***w*** (for continuous variables) and ***v*** (for ordinal variables). A different symbol is used since the univariate response vector can be either continuous or ordinal. In the electronic supplementary material (e.g. §1.5), the symbol ***y*** is also used for a vector of mixed responses for a single individual. We consider this ‘abuse of notation’ acceptable since the way the symbol is used is always clear from the context.

**Figure 2.**
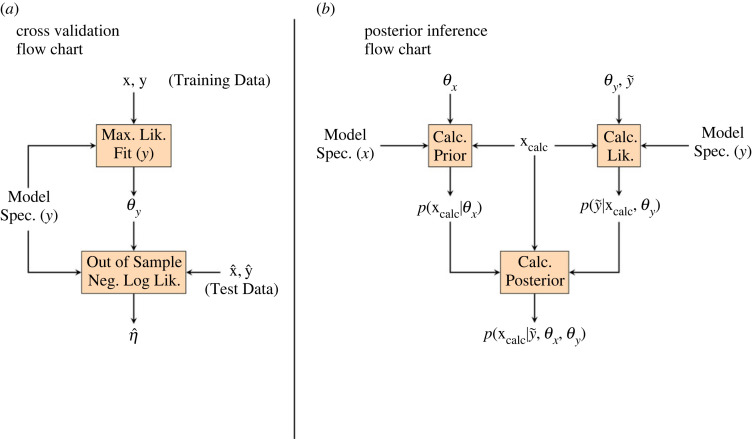
(*a*) The flow chart summarizes the cross-validation for a univariate model. The vectors ***x*** and ***y*** are the training data and the vectors $\hat{x}$ and $\hat{y}$ the test data. For each fit, a model specification is needed. This consists primarily of the mean function and the noise mode (see electronic supplementary material). The metric for choosing among models is the out-of-sample negative log-likelihood calculated using the test data. (*b*) The flow chart summarizes the posterior calculation. The vector parametrizes the prior, and the vector, which results from a maximum-likelihood fit as shown in the left flow chart, parametrizes the likelihood. Although we use an offset Weibull mixture for the specification of the prior (‘Model Spec. (*x*)’), yada supports additional specifications that are more suitable for archaeological and forensic age estimation (notably a uniform prior). A vector of ages at which to calculate the posterior (calc) must be provided (though yada provides tools for automatically choosing this vector). The response vector represents the new response variable for which the posterior density (a function of age, calc) is calculated. The prior and likelihood are multiplied pointwise to yield an un-normalized posterior, which is then normalized to integrate to 1, yielding the final posterior.

The model specification consists of a specification of the mean, a specification of the noise and (for ordinal variables) the number of ordinal categories for ordinal variables, *M*. For continuous variables, we allow only a single specification of the mean (power law) and two specifications of the noise (constant and linear positive intercept), so the cross-validation for each continuous variable includes two models. A maximum-likelihood fit (full details in the electronic supplementary material) is done using the training data for each cross-validation fold, yielding a best-fit parameter vector, $\theta_{y}$. This parameter vector, the model specification and the test data are used to calculate the out-of-sample negative log-likelihood for the fold, $\hat{\eta }$; the overall out-of-sample negative log-likelihood is then calculated by summing those from the four cross-validation folds. The preferred model is the one with the lowest value of this summed negative log-likelihood (figure 2*a*). The negative log-likelihood was selected for model selection because of consistency in research design; for example, negative log-likelihood was used for optimization.

#### 2.1.4. Multivariate mixed models and conditional dependence

As discussed above, few previous approaches have adequately allowed for the simultaneous use of ordinal and continuous variables to do posterior inference. The MCP accommodates mixed variables by assuming that the only difference between ordinal and continuous variables is that the latent response for ordinal variables cannot be directly observed. One way to form a multivariate model is to ‘stack’ all the univariate models—that is, to form the likelihood by pointwise multiplying the likelihoods of all the individual univariate models. Below, we describe how to generalize this approach to allow conditional dependence among variables. If all the correlation terms in the covariance matrix are 0, the conditionally independent model obtained via ‘stacking’ is exactly recovered.

Variables are statistically independent if the value (or realization) of one variable does not influence the probability of the other. Two variables are conditionally dependent if after conditioning on another variable, they are not conditionally independent of each other. For example, individuals who have one long bone measurement that is large for their age (age is the conditioned variable) will frequently have large values for other long bone measurements. If this is the case, and conditional independence is nevertheless assumed, posterior inference will be overconfident, which means the posterior density function will be too narrow. The reason is that, when assuming conditional independence, one assumes that each variable independently informs on the posterior age distribution. Yet, a variable that is perfectly correlated with another after conditioning provides no additional information.

For mixed models, the mean response is modelled independently for each individual variable as described above for univariate models; however, the noise is modelled as a multivariate normal with a covariance matrix, *Σ*. The scale of the noise is exactly as before for univariate models. For example, for the *j*th ordinal variable, the corresponding diagonal term of the covariance matrix is $\Sigma_{\;jj}=\gamma_{j}^{2}$ (and similarly for continuous variables). In principle, we allow arbitrary values for the off-diagonal terms, which we parametrize using correlation coefficients *ρ_il_*, $\Sigma_{il}=\rho_{il}\Sigma_{ii}\Sigma_{ll}$. However, calculating the likelihood for a multivariate model (see electronic supplementary material for full details) involves integrating a multivariate normal density on a rectangular domain, which is computationally expensive. For example, the six-variable model used in this example took approximately 5 days. As you increase the number of variables, the processing time increases. Therefore, it is useful to group together variables with similar conditional dependencies and assign to them the same values for the correlation coefficients. In so doing, the parametrization for the correlation terms involves fewer terms, and we choose to represent these terms using the parameter vector ***z***. See the electronic supplementary material for the precise mapping between the elements of ***z*** and the correlation coefficients, *ρ_il_*.

Adopting such groupings does not reduce the time needed for the likelihood calculation, but it does reduce the dimensionality of the optimization problem to find maximum-likelihood parameter vectors. Unlike the overall scale of the noise, we do not allow the correlation coefficients to depend on *x* (in this case, age), an assumption that could be relaxed in future work.

#### 2.1.5. Cross-validation of conditional dependence (Step 2)

The second cross-validation step involved comparing a conditionally independent model, in which all the correlation coefficients (*ρ_il_*) were set to zero, with a conditionally dependent model, in which they were varied, along with the other parameters, to maximize the likelihood. In fact, the likelihood of the conditionally independent model is simply the pointwise product of the likelihoods of the univariate models, which are statistically independent of each other by construction. The parameter values of the multivariate conditionally independent model are exactly those of the univariate fits. Essentially, the conditionally independent model can be constructed directly from the univariate fits. We restricted the correlation terms in the matter outlined in the preceding paragraph by requiring that the dental variables (max_M1 and man_I2) behaved identically and that the continuous variables (FDL and RDL) behaved identically. This yielded eight independent correlation terms (see electronic supplementary material). Similar to univariate cross-validation (Step 1), fourfold cross-validation is used to calculate summed negative log-likelihood estimates for the conditionally independent and conditionally dependent models, of which the lowest summed estimate is the preferred model.

#### 2.1.6. Missing values

A major advantage of the mixed model just described is that it can inherently accommodate missing values for any response variable, since missing variables can be accounted for by marginalizing them—that is, integrating them on the interval negative to positive infinity. For the likelihood calculation, marginalizing a variable is exactly equivalent to removing that variable from the likelihood calculation.

#### 2.1.7. Posterior inference, information gain and severity of model mis-specification

Here, we describe how posterior inference is conducted, explain how the KL divergence can be used to quantify information gain in going from a prior to posterior density, and use the KL divergence to quantify the severity of model specification with several examples. We provide a brief description of the procedure for posterior inference, but full details are available in electronic supplementary material, §1.5 and the process is summarized in figure 2*b*. The goal of this data-driven example is to estimate age using a set of response variables. We start with a prior probability density over age. In practice, this is often assumed to be a minimally informative distribution (probably a uniform distribution) or, for skeletal age estimation, an informative distribution, such as late twentieth-century homicides in the United States (e.g. [64]). We use an offset Weibull mixture fit of the vector of known ages of individuals in the SVAD database (see electronic supplementary material, figure S1). The likelihood for the Bayesian update step is the likelihood of the known response vector as a function of age. The prior and likelihood are multiplied pointwise for each *x*-value, which yields an un-normalized posterior density vector. This vector is normalized to numerically integrate to 1, which yields the final posterior. In figure 2, $\tilde{y}$ consists of a mixed set of responses for a single individual, which, as already discussed above, could be considered abuse of notation. This vector $\tilde{y}$ provides the additional information used to update the prior density, yielding the posterior density. We use the 2.5% and 97.5% values of the quantile of the posterior distribution (i.e. equal-tailed interval) rather than the highest density interval to determine the confidence interval and we used the posterior mean as the point estimate. The KL divergence of the posterior distribution *P*(*x*) from the prior distribution *Q*(*x*) is

$$D_{\mathrm{KL}}=-\underset{n}{\sum }P(x_{n})\log_{2}\frac{Q(x_{n})}{P(x_{n})},$$

where ***x*** is a vector of independent variables (i.e. ages) indexed by *n* where the quantities of interest are calculated (usually a regularly spaced set of values across the entire domain of interest). We use a logarithm of base 2 so that the unit of the KL divergence is bits. The KL divergence measures the information gain in going from the prior to posterior—that is, it quantifies the value of knowing the response vector, $\tilde{y}$. The larger the value, the more information has been gained.

## 3. Results

### 3.1. Univariate models

Table 1 summarizes the cross-validation results for univariate ordinal and continuous models. For ordinal variables, we allow three specifications of the mean (power law ordinal, logarithmic and linear) and the same two noise specifications as for continuous variables, so the cross-validation for each ordinal variable involves six models. The power law was chosen for all six variables, regardless of being ordinal or continuous. The heteroscedastic model had the smallest negative log-likelihoods for the two continuous variables and three of the four ordinal variables, indicating that for five of the six variables the more complex heteroscedastic model was preferred to the homoscedastic model (table 1). These results demonstrate that, for this data, heteroscedasticity and nonlinearity must be accounted for in the statistical modelling for each variable; failure to do so leads to poor, mis-specified models.

### 3.2. Multivariate mixed models

We fit a conditionally dependent model to the full set of six variables and found that, indeed, conditional correlations exist. The conditional correlations are captured by the *z*-parameters in electronic supplementary material, §1.4. We used the KL divergence to quantify the effect of incorrectly assuming conditional dependence. We used the same cross-validation folds for the second cross-validation step as for the first. The out-of-sample negative log-likelihood for the conditionally independent six-variable model was 5818.61, whereas that of the conditionally dependent six-variable model was 5534.68. Since the latter is smaller, the conditionally dependent mode is preferred.

**Figure 3.**
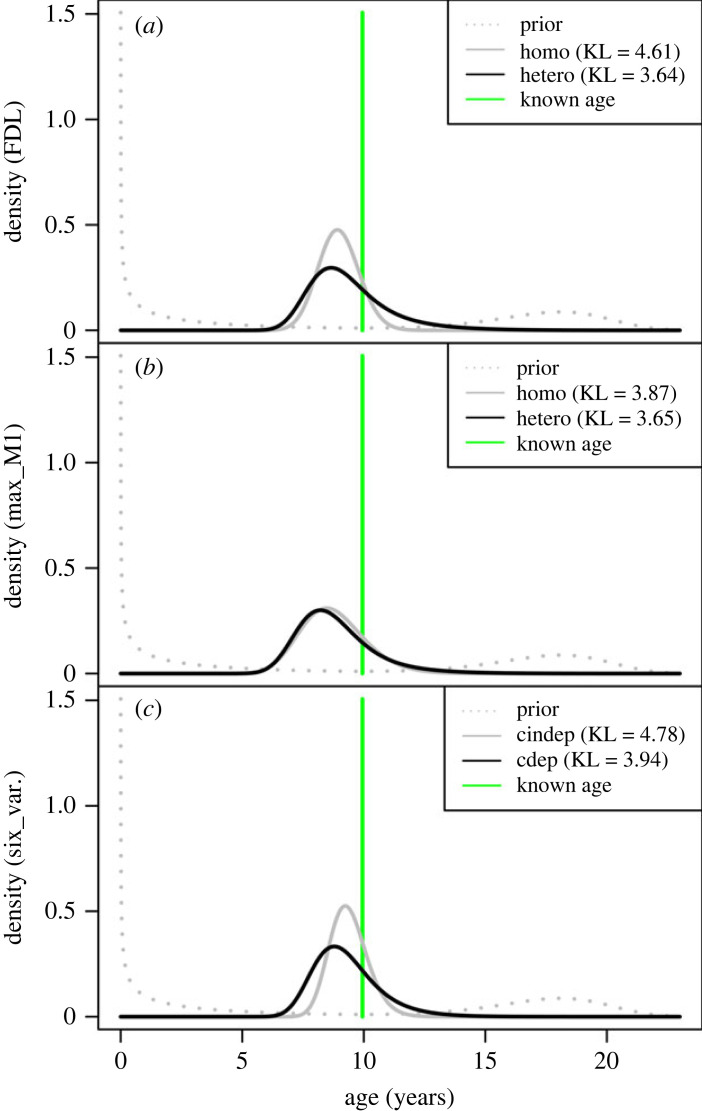
Posterior density estimates for a variety of models for an individual of known age (the vertical green bars). The plots share the same *x*-axis and *y*-axis ranges. The legend provides the KL divergence values for each of two models, one bad (mis-specified) and one good. (*a*) Posterior density estimate using only the FDL response. The mis-specified homoscedastic model is over confident, which is reflected by a larger value for the KL divergence (4.76 versus 3.73). (*b*) Posterior density estimate using only the max_M1 response. The mis-specified homoscedastic model is slightly over confident. (*c*) Posterior density estimate using all six variables. The mis-specified conditionally independent model is over confident.

Figure 3 presents three examples of posterior inference, in each case involving the ‘good’ model and a ‘poor’ or mis-specified model. All three plots share the same *x*-axis, have the same *y*-axis range, and are for an individual with a response vector $y=[1,5,10,11,317.11,167.5{]}^{T}$, where the variable ordering is ‘HME_EF’, ‘TC_Oss’, ‘max_M1’, ‘man_I2’, ‘FDL’ (mm) and ‘RDL’ (mm). In the vector, ‘1’ corresponds to a developmental score of ‘present’ for the HME_EF, ‘5’ corresponds to the ‘present’ tarsal count, and so forth. The individual has a known age of *x* = 9.945205 years. Figure 3*a* uses only the FDL value for posterior inference. As discussed above, the cross-validation favours the heteroscedastic model, so it is the specified model (i.e. the good model), and the homoscedastic model is the mis-specified model. The reconstructed uncertainty for a variable that exhibits heteroscedasticity is usually too high (for young individuals) or too low (for old individuals) if a homoscedastic model is incorrectly used. In figure 3*a*, the individual is relatively old, so the homoscedastic is overconfident, and that is quantified by the KL divergence, which is 4.61 bits for the homoscedastic FDL model and 3.64 bits for the heteroscedastic FDL model.

For some observations in an intermediate range, or if the amount of heteroscedasticity is small, the heteroscedastic model and mis-specified homoscedastic model can yield similar uncertainties. This is illustrated in figure 3*b*, which shows the posterior density if only the max_M1 (*v* = 10) is used for posterior inference. The KL divergence is 3.87 for the homoscedastic max_M1 model and 3.65 for the heteroscedastic max_M1 model. Whereas the homoscedastic FDL model is 0.97 bits too confident, the homoscedastic max_M1 model is only 0.22 bits too confident.

Figure 3*c* compares the posterior density of the good, conditionally dependent, six-variable model with the mis-specified (overly confident), six-variable, conditionally independent model. The KL divergence for the conditionally independent model is 4.78 and that of the conditionally dependent model is 3.94, with the former being 0.84 bits too confident. To calculate the KL divergence, one must specify the response vector to use for posterior inference. In the electronic supplementary material §4, we describe how one can instead treat the response vector as a random variable (conditioning on a baseline age). This yields an alternative information theoretic measure, the mutual information, which indicates on average how much information is gained, at each age, from learning the response vector. In the electronic supplementary material, figure S3 visualizes the mutual information of FDL as a continuous measure and FDL as an ordinal measure. The plot demonstrates that information is necessarily lost when turning the continuous FDL measurement into an ordinal measure.

## 4. Discussion and conclusion

To the authors’ knowledge, the MCP is the only cumulative link-generalized linear TA algorithm that accommodates heteroscedasticity, a mix of ordinal and continuous response variables, missing values, conditional dependence and alternative specifications of the mean and noise response functions. The model supports, in principle, any number of variables and any numbers of categories for each ordinal variable. We allow only one independent variable (demonstrated here with age) and use a specific parametric form for the dependence of latent dependent variables on the independent variable. This combination of features into one model is primarily what makes it so unique; other models may incorporate a number of these features, but none combine all of them. Therefore, while the MCP was developmentally driven by the needs of subadult age estimation, it has a wide range of potential applications. Specifically, the MCP algorithm is useful in any situation where the predictor variables are continuous and/or ordinal, the response variable is continuous, the predictor variables' relationship to the response variable is nonlinear and/or linear and homoscedastic and/or heteroscedastic, and—if using multiple variables—conditional dependence can be incorporated. This combination easily extends the algorithm's application beyond the boundaries of biological anthropology, including scaling, behavioural ecology and forensic sciences, among others.

Information theory (the KL divergence and mutual information) can be used to assess information gain when doing posterior inference, including quantifying the impact of a mis-specified model. The reason we can identify mis-specified models is that we conducted model selection using fourfold cross-validation. This contrasts with much, though certainly not all, previous work in biological anthropology, where no checks on the validity of core modelling assumptions were done. As Gelman & Shalizi [65, p. 10] point out, however, ‘all models in use are wrong—not merely falsifiable, but actually false’. The implication of this observation is that there may be better models than the ones we assessed in the cross-validation. Indeed, it seems likely that one assumption we made will not hold up to future scrutiny in biological anthropology in particular: the assumption that the conditional correlations (the *z*-parameters described above and in electronic supplementary material, §1.4) do not depend on *x*, which in this example is age. That is, while we allow for the possibility that the mean response and diagonal terms of the multivariate covariate matrix are age dependent, we do not allow for age dependence in the correlations, which contradicts preliminary findings we report in Stull *et al*. [60]. In assessing the presence of conditional correlations, we only tested a four-group model (conditionally dependent model) for comparison against a conditionally independent model. Second, we already know that future work should assess the time-dependence of the conditional correlation parameters, so any effort put into assessing groupings of non-age-dependent correlations may be wasted. In short, we acknowledge these limitations in our modelling, but think it is better to report our findings in their current manifestation before tackling these challenging complications.

Scientific integrity depends on the reproducibility of research, which is achieved through transparency [66]. Anthropologists are recognizing the importance of an ecosystem that recognizes reproducibility and transparency as an ethical responsibility and a scientific obligation (e.g. [67,68]); however, restricted access to algorithms and data have hampered innovation and the widespread use of state-of-the-art algorithms, especially for age estimation in biological anthropology. Our work is fully reproducible following the source code and data available via open access (https://github.com/MichaelHoltonPrice/rsos_mcp_intro) and the full algorithm is documented in the electronic supplementary material. The yada package (GitHub.com/MichaelHoltonPrice/yada) contains all the original functions used in the MCP pipeline.

While reproducibility is essential, one of the largest disconnects in biological anthropology is between researchers and practitioners. As such, innovative contributions like the MCP, have a risk of minimally impacting the larger scientific community because of perceived or real limitations to their application both within the anthropological realm and in other research contexts. Scientists have different niches, expertise, backgrounds and, therefore, the capability of each to use computationally demanding statistical models. ‘Better models’ are theoretically preferred, but if there is no ‘easy’ way to apply them (i.e. through graphical user interfaces (GUIs)), then they will not be applied. Thus, accessibility is crucial for methodological and other overall advancements in any field. While advancing the overall scientific endeavours, accessibility concurrently lessens the learning curve associated with a complex analytical approach, and increases the implementation of appropriate statistics, no matter the economic means of the practitioner or the population one is serving [69]. Therefore, a step-by-step vignette (RPubs.com/elainechu/mcp_vignette) provides users with an easy-to-follow format to fully reproduce the MCP models discussed in this article. Additionally in the vignette, we have provided an R script for easy-to-use code for future researchers interested in applying the MCP to their own research questions.

As we have learned in preparing this manuscript, building and fitting models that can accommodate the complexity of biological data can be quite challenging. Hindrances to innovation are therefore linked to computational limitations, a prevailing culture of restricted access to data, and limited knowledge and skills in the biological anthropology community for designing novel algorithms. There are, of course, some anthropologists (e.g. Dr. Lyle Konigsberg) who make their code freely available, and we applaud for his transparency and openness. While these anthropologists have set the precedent, fully reproducible research has not been the prevailing practice in biological anthropology [70]. Active collaboration to develop and improve open-source code is, in our minds, the only answer to the modelling challenges faced by biological anthropologists working with inherently complex biological data and forensic scientists needing advanced analytical techniques.

## Data Availability

The data and analyses are all freely available. The data used in the current study are available in the Zenodo Subadult Virtual Anthropology Database Community: https://doi.org/10.5281/zenodo.5193208 [71]. The vignette is freely available here: https://rpubs.com/elainechu/mcp_vignette. The relevant code for this work is stored in GitHub: https://github.com/michaelholtonprice/rsos_mcp_intro and has been archived within the Zenodo repository: https://doi.org/10.5281/zenodo.7603754 [72]. The data are provided in the electronic supplementary material [73].

## References

[1] Boldsen J, Milner G, Konigsberg L, Wood J. 2002 Transition analysis: a new method for estimating age from skeletons. In Paleodemography: age distributions from skeletal samples (eds R Hoppa, J Vaupel), pp. 73-106. Cambridge, UK: Cambrige University Press.

[2] Jackman S. 2009 Bayesian analysis for the social sciences. New York, NY: Wiley and Sons.

[3] Konigsberg L, Herrmann N, Wescott D, Kimmerle E. 2008 Estimation and evidence in forensic anthropology: age-at-death. J. Forensic Sci. **53**, 541-557. (10.1111/j.1556-4029.2008.00710.x) 18471197

[4] McElreath R. 2015 Statistical rethinking: a Bayesian course with examples in R and stan [internet]. Boca Raton, FL: CRC Press LLC. See http://ebookcentral.proquest.com/lib/knowledgecenter/detail.action?docID=4648054 (accessed 16 April 2022).

[5] McKelvey RD, Zavoina W. 1975 A statistical model for the analysis of ordinal level dependent variables. J. Math. Sociol. **4**, 103-120. (10.1080/0022250X.1975.9989847)

[6] Langley-Shirley N, Jantz RL. 2010 A Bayesian approach to age estimation in modern Americans from the clavicle. J. Forensic Sci. **55**, 571-583. (10.1111/j.1556-4029.2010.01089.x) 20384935

[7] Lottering N, MacGregor DM, Alston CL, Gregory LS. 2014 Ontogeny of the spheno-occipital synchondrosis in a modern Queensland, Australian population using computed tomography. Am. J. Phys. Anthropol. **157**, 42-57. (10.1002/ajpa.22687) 25546173

[8] Shirley N, Jantz RL. 2011 Spheno-occipital synchondrosis fusion in modern Americans. J. Forensic Sci. **56**, 580-585. (10.1111/j.1556-4029.2011.01705.x) 21361933

[9] Getz SM. 2020 The use of transition analysis in skeletal age estimation. WIREs Forensic Sci. **2**, e1378. (10.1002/wfs2.1378)

[10] Konigsberg L, Frankenberg S, Walker R. 1997 Regress what on what? Paleodemographic age esitmation as a calibration problem. In Integrating archaeological demography: multidisciplinary approaches to prehistoric population (ed. R Paine), Occasional Paper 24, pp. 64-88. Carbondale, IL: Southern Illinois University Center for Archaeological Investigations.

[11] Konigsberg LW, Frankenberg SR. 1992 Estimation of age structure in anthropological demography. Am. J. Phys. Anthropol. **89**, 235-256. (10.1002/ajpa.1330890208) 1443096

[12] Nikita E, Nikitas P. 2019 Skeletal age-at-death estimation: Bayesian versus regression methods. Forensic Sci. Int. **297**, 56. (10.1016/j.forsciint.2019.01.033) 30776778

[13] Steadman DW, Adams BJ, Konigsberg LW. 2006 Statistical basis for positive identification in forensic anthropology. Am. J. Phys. Anthropol. **131**, 15-26. (10.1002/ajpa.20393) 16485302

[14] Evett I. 2015 The logical foundations of forensic science: towards reliable knowledge. Phil. Trans. R. Soc. Lond. B **370**, 20140263. (10.1098/rstb.2014.0263) 26101288 PMC4581007

[15] Evett IW. 1987 Bayesian inference and forensic science: problems and perspectives. J. R. Stat. Soc. Ser. D **36**, 99-105.

[16] Konigsberg L, Holman D. 1999 Estimation of age at death from dental emergence and implications for studies of prehistoric somatic growth. In Human growth in the past: studies from bones and teeth (eds R Hoppa, C Fitzgerald), pp. 264-289. Cambridge, UK: Cambridge University Press.

[17] Konigsberg LW, Frankenberg SR. 2013 Bayes in biological anthropology. Am. J. Phys. Anthropol. **152**, 153-184. (10.1002/ajpa.22397) 24190509

[18] Mostad P, Schmeling A, Tamsen F. 2022 Mathematically optimal decisions in forensic age assessment. Int. J. Legal Med. **136**, 765-776. (10.1007/s00414-021-02749-y) 34910231 PMC9005397

[19] Sironi E, Vuille J, Morling N, Taroni F. 2017 On the Bayesian approach to forensic age estimation of living individuals. Forensic Sci. Int. **281**, e24-e29. (10.1016/j.forsciint.2017.11.007) 29162298

[20] Taroni F, Bozza S, Biedermann A, Garbolino P, Aitken C. 2010 Data analysis in forensic science: a Bayesian decision perspective. New York, NY: John Wiley & Sons.

[21] Kim J, Algee-Hewitt BFB, Konigsberg LW. 2019 Inferring age at death for Japanese and Thai skeletal samples under a Bayesian framework of analysis: a test of priors and their effects on estimation. Forensic Anthropol. **2**, 273-292.

[22] Hoppa RD, Vaupel JW. 2002 Paleodemography: age distributions from skeletal samples. Cambridge, UK: Cambridge University Press.

[23] Sironi E, Gallidabino M, Weyermann C, Taroni F. 2016 Probabilistic graphical models to deal with age estimation of living persons. Int. J. Legal Med. **130**, 475-488. (10.1007/s00414-015-1173-7) 25794687

[24] Konigsberg LW. 2015 Multivariate cumulative probit for age estimation using ordinal categorical data. Ann. Hum. Biol. **42**, 368-378. (10.3109/03014460.2015.1045430) 26190374

[25] Bullock M, Márquez L, Hernández P, Ruíz F. 2013 Paleodemographic age-at-death distributions of two Mexican skeletal collections: a comparison of transition analysis and traditional aging methods. Am. J. Phys. Anthropol. **152**, 67-78. (10.1002/ajpa.22329) 24037796

[26] DiGangi E, Bethard J, Kimmerle E, Konigsberg L. 2009 A new method for estimating age-at-death from the first rib. Am. J. Phys. Anthropol. **138**, 164-176. (10.1002/ajpa.20916) 18711740

[27] Godde K. 2017 The use of informative priors in Bayesian modeling age-at-death; a quick look at chronological and biological age changes in the sacroiliac joint in American males. AIMS Public Health. **4**, 278-288. (10.3934/publichealth.2017.3.278) 29546217 PMC5690454

[28] Godde K, Hens SM. 2015 Modeling senescence changes of the pubic symphysis in historic Italian populations: a comparison of the Rostock and forensic approaches to aging using transition analysis. Am. J. Phys. Anthropol. **156**, 466-473. (10.1002/ajpa.22671) 25407762

[29] Godde K, Hens SM. 2021 An epidemiological approach to the analysis of cribra orbitalia as an indicator of health status and mortality in medieval and post-medieval London under a model of parasitic infection. Am. J. Phys. Anthropol. **174**, 631-645. (10.1002/ajpa.24244) 33528042

[30] Hens SM, Godde K. 2016 Auricular surface aging: comparing two methods that assess morphological change in the ilium with Bayesian analyses. J. Forensic Sci. **61**, S30-S38. (10.1111/1556-4029.12982) 27405023

[31] Lucy D, Aykroyd RG, Pollard AM, Solheim T. 1996 A Bayesian approach to adult human age estimation from dental observations by Johanson's age changes. J. Forensic Sci. **41**, 189-194. (10.1520/JFS15411J) 8871375

[32] Nikita E, Xanthopoulou P, Kranioti E. 2018 An evaluation of Bayesian age estimation using the auricular surface in modern Greek material. Forensic Sci. Int. **291**, 1-11. (10.1016/j.forsciint.2018.07.029) 30118876

[33] Prince DA, Kimmerle EH, Konigsberg LW. 2008 A Bayesian approach to estimate skeletal age-at-death utilizing dental wear. J. Forensic Sci. **53**, 588-593. (10.1111/j.1556-4029.2008.00714.x) 18471201

[34] Tangmose S, Thevissen P, Lynnerup N, Willems G, Boldsen J. 2015 Age estimation in the living: transition analysis on developing third molars. Forensic Sci. Int. **257**, 512.e1-512.e7. (10.1016/j.forsciint.2015.07.049) 26342939

[35] De Tobel J et al. 2020 Multi-factorial age estimation: a Bayesian approach combining dental and skeletal magnetic resonance imaging. Forensic Sci. Int. **306**, 110054. (10.1016/j.forsciint.2019.110054) 31778924

[36] Godde K, Pasillas V, Sanchez A. 2020 Survival analysis of the Black Death: social inequality of women and the perils of life and death in medieval London. Am. J. Phys. Anthropol. **173**, 168-178. (10.1002/ajpa.24081) 32472637

[37] Gunst K, Mesotten K, Carbonez A, Willems G. 2003 Third molar root development in relation to chronological age: a large sample sized retrospective study. Forensic Sci. Int. **136**, 52-57. (10.1016/S0379-0738(03)00263-9) 12969620

[38] Konigsberg LW, Frankenberg SR, Sgheiza V, Liversidge HM. 2022 Prior probabilities and the age threshold problem: first and second molar development. Hum. Biol. **93**, 51-63. (10.13110/humanbiology.93.1.02) 35338702

[39] Kumagai A, Willems G, Franco A, Thevissen P. 2018 Age estimation combining radiographic information of two dental and four skeletal predictors in children and subadults. Int. J. Legal Med. **132**, 1769-1777. (10.1007/s00414-018-1910-9) 30099588

[40] Štern D, Payer C, Giuliani N, Urschler M. 2019 Automatic age estimation and majority age classification from multi-factorial MRI data. IEEE J. Biomed. Health Informatics **23**, 1392-1403. (10.1109/JBHI.2018.2869606) 31059459

[41] Thevissen PW, Fieuws S, Willems G. 2010 Human dental age estimation using third molar developmental stages: does a Bayesian approach outperform regression models to discriminate between juveniles and adults? Int. J. Legal Med. **124**, 35-42. (10.1007/s00414-009-0329-8) 19238421

[42] Gueorguieva RV, Agresti A. 2001 A correlated probit model for joint modeling of clustered binary and continuous responses. J. Am. Stat. Assoc. **96**, 1102-1112. (10.1198/016214501753208762)

[43] Stull KE, L'Abbé EN, Ousley SD. 2014 Using multivariate adaptive regression splines to estimate subadult age from diaphyseal dimensions. Am. J. Phys. Anthropol. **154**, 376-386. (10.1002/ajpa.22522) 24782395

[44] Corron L, Marchal F, Condemi S, Chaumoître K, Adalian P. 2017 A new approach of juvenile age estimation using measurements of the ilium and multivariate adaptive regression splines (MARS) models for better age prediction. J. Forensic Sci. **62**, 18-29. (10.1111/1556-4029.13224) 27792240

[45] Corron L, Marchal F, Condemi S, Telmon S. 2019 Integrating growth variability of the ilium, fifth lumbar vertebra, and clavicle with multivariate adaptive regression splines models for subadult age estimation. J. Forensic Sci. **64**, 34. (10.1111/1556-4029.13831) 29852519

[46] Buikstra JE, Konigsberg LW. 1985 Paleodemography: critiques and controversies. Am. Anthropol. **87**, 316-333. (10.1525/aa.1985.87.2.02a00050)

[47] Buschang P. 1982 Differential long bone growth of children between two months and eleven years. Am. J. Phys. Anthropol. **58**, 291-295. (10.1002/ajpa.1330580307) 7124922

[48] Cardoso HFV, Abrantes J, Humphrey L. 2013 Age estimation of immature human skeletal remains from the diaphyseal length of the long bones in the postnatal period. Int. J. Legal Med. **162**, 19-35. 10.1007/s00414-013-0925-5 24126574

[49] Cardoso HFV, Vandergugten J, Humphrey L. 2017 Age estimation of immature human skeletal remains from the metaphyseal and epiphyseal widths of the long bones in the post-natal period. Am. J. Phys. Anthropol. **162**, 19-35. (10.1002/ajpa.23081) 27613447

[50] Frelat MA, Mittereocker P. 2011 Postnatal ontogeny of tibia and femur form in two human populations: a multivariate morphometric analysis. Am. J. Hum. Biol. **23**, 796-804. (10.1002/ajhb.21217) 21957036

[51] Hoppa RD. 2000 Population variation in osteological aging criteria: an example from the pubic symphysis. Am. J. Phys. Anthropol. **111**, 185-191. (10.1002/(SICI)1096-8644(200002)111:2<185::AID-AJPA5>3.0.CO;2-4) 10640946

[52] Hoppa RD. 2000 What to do with long bones: toward a progressive paleoauxology. Anthropologie (1962-) **38**, 23-32.

[53] Humphrey LT. 1998 Growth patterns in the modern human skeleton. Am. J. Phys. Anthropol. **105**, 57-72. (10.1002/(SICI)1096-8644(199801)105:1<57::AID-AJPA6>3.0.CO;2-A) 9537928

[54] Rissech C, López-Costas O, Turbón D. 2013 Humeral development from neonatal period to skeletal maturity—application in age and sex assessment. Int. J. Legal Med. **127**, 201-212. (10.1007/s00414-012-0713-7) 22588220

[55] Smith S. 2007 Stature estimation of 3–10 year-old children from long bone lengths. J. Forensic Sci. **52**, 538-546. (10.1111/j.1556-4029.2007.00428.x) 17456079

[56] Feng C et al. 2014 Log-transformation and its implications for data analysis. Shanghai Arch Psychiatry **26**, 105-109. 25092958 10.3969/j.issn.1002-0829.2014.02.009 PMC4120293

[57] Kaufman R. 2013 Heteroskedasticity in regression: detection and correction. Thousand Oaks, CA: SAGE Publications, Inc. See https://methods.sagepub.com/book/heteroskedasticity-in-regression

[58] Stull KE, Corron LK. 2022 The subadult virtual anthropology database (SVAD): an accessible repository of contemporary subadult reference data. Forensic Sci. **2**, 20-36. (10.3390/forensicsci2010003)

[59] Stull K, Corron LK. 2021 Code for: Subadult Virtual Anthropology Database (SVAD) data collection protocol: Amira. Zenodo. (10.5281/zenodo.5348411)

[60] Stull K, Corron LK. 2021 Code for: Subadult Virtual Anthropology Database (SVAD) data collection protocol: epiphyseal fusion, diaphyseal dimensions, dental development stages, vertebral neural canal dimensions. Zenodo. (10.5281/zenodo.5348392)

[61] AlQahtani SJ, Hector MP, Liversidge HM. 2010 Brief communication: the London atlas of human tooth development and eruption. Am. J. Phys. Anthropol. **142**, 481-490. (10.1002/ajpa.21258) 20310064

[62] Corron LK, Stock MK, Cole SJ, Hulse CN, Garvin HM, Klales AR, Stull KE. 2021 Standardizing ordinal subadult age indicators: testing for observer agreement and consistency across modalities. Forensic Sci. Int. **320**, 110687. (10.1016/j.forsciint.2021.110687) 33461006

[63] R Core Team. 2019 R: a language and environment for statistical computing. Vienna, Austria: R Foundation for Statistical Computing. See https://www.R-project.org/

[64] Milner GR, Boldsen JL. 2012 Transition analysis: a validation study with known-age modern American skeletons. Am. J. Phys. Anthropol. **148**, 98-110. (10.1002/ajpa.22047) 22419394

[65] Gelman A, Shalizi CR. 2013 Philosophy and the practice of Bayesian statistics. Br. J. Math. Stat. Psychol. **66**, 8-38. (10.1111/j.2044-8317.2011.02037.x) 22364575 PMC4476974

[66] Kretser A et al. 2019 Scientific integrity principles and best practices: recommendations from a Scientific Integrity Consortium. Sci. Eng. Ethics **25**, 327-355. (10.1007/s11948-019-00094-3) 30810892 PMC6450850

[67] Beheim B. 2016 Reproducible research as our new default. Anthropol. News **57**, e57. (10.1111/j.1556-3502.2016.570523.x)

[68] Martin MA. 2019 Biological anthropology in 2018: grounded in theory, questioning contexts, embracing innovation. Am. Anthropol. **121**, 417-430. (10.1111/aman.13233)

[69] Hens SM, Godde K. 2020 New approaches to age estimation using palatal suture fusion. J. Forensic Sci. **65**, 1406-1415. (10.1111/1556-4029.14485) 32557604

[70] Tennant JP, Waldner F, Jacques DC, Masuzzo P, Collister LB, Hartgerink CHJ. 2016 The academic, economic and societal impacts of Open Access: an evidence-based review. F1000Research **5**, 632. (10.12688/f1000research.8460.3) 27158456 PMC4837983

[71] Stull KE, Chu EY, Corron LK, Price MH. 2023 Data from: Mixed cumulative probit: a multivariate generalization of transition analysis that accommodates variation in the shape, spread and structure of data. Zenodo. (10.5281/zenodo.5193208) PMC9974299 36866077

[72] Stull KE, Chu EY, Corron LK, Price MH. 2023 Data from: Mixed cumulative probit: a multivariate generalization of transition analysis that accommodates variation in the shape, spread and structure of data. Zenodo. (10.5281/zenodo.7603754) PMC9974299 36866077

[73] Stull KE, Chu EY, Corron LK, Price MH. 2023 Mixed cumulative probit: a multivariate generalization of transition analysis that accommodates variation in the shape, spread and structure of data. Figshare. (10.6084/m9.figshare.c.6431688) PMC9974299 36866077

